# Corrosion Resistance of Inconel 625 CMT-Cladded Layers after Long-Term Exposure to Biomass and Waste Ashes in High-Temperature Conversion Processes

**DOI:** 10.3390/ma13194374

**Published:** 2020-10-01

**Authors:** Aleksandra Błoniarz, Marcus Schreiner, Markus Reinmöller, Agnieszka Kopia

**Affiliations:** 1 Department of Surface Engineering and Materials Characterisation, AGH University of Science and Technology, A. Mickiewicza 30, 30-059 Krakow, Poland; debovska@agh.edu.pl; 2 Institute of Energy Process Engineering and Chemical Engineering (IEC), Technische Universität Bergakademie Freiberg, Fuchsmühlenweg 9 D, 09599 Freiberg, Germany; Marcus.Schreiner@iec.tu-freiberg.de (M.S.); Markus.Reinmoeller@iec.tu-freiberg.de (M.R.); 3 Fraunhofer Institute for Microstructure of Materials and Systems (IMWS), Walter-Hülse-Str. 1, 06120 Halle, Germany; 4 Center of Innovation Competence (CIC) Virtuhcon, Fuchsmühlenweg 9 D, 09599 Freiberg, Germany

**Keywords:** Inconel 625-coated boiler steel, cold metal transfer (CMT), biomass, waste, ash, high-temperature corrosion, thermochemical conversion processes

## Abstract

The present study investigated the effect of corrosion on an Inconel 625-cladded layer using the cold metal transfer (CMT) method. The corrosion was caused by various ashes and high process temperatures. The ashes were obtained from the biomasses of mixed wood and oat straw, as well as from sewage sludge, by ashing. Long-term corrosion tests were carried out at 650 °C over a period of 1000 h. The chemical composition, mineral phases, and corrosion effects were studied by X-ray fluorescence (XRF), scanning electron microscopy equipped with energy-dispersive X-rays (SEM–EDX), and X-ray diffraction (XRD) from the surface and on the cross-section of the samples. The chemical composition of the ashes was quite different, but representative of their particular fuel. Together with the effects of the operating temperature and mass transfer, significant differences in the degree of the corrosion depth were detected for the various ashes. For the investigated samples, the corrosion mechanisms were inferred based on the identified corrosion products.

## 1. Introduction

The energy supply is a crucial issue for the economy. Obtaining energy is not only associated with economic benefits for the state on an international level, but also directly affects comfort and quality of life among the inhabitants of a country. However, the energy consumption of most countries is currently based on fossil fuels, thus an increase in energy use may not only cause significant problems to natural ecosystems, but may also impact people’s life and health [1,2]. There is a social, political, and economic consensus that the global warming accelerated by CO_2_ emissions and other negative effects of fossil fuels should be reduced; in consequence, investment in technologies for tapping renewable energy resources is increasing. For example, the European Union has set ambitious goals until 2030 regarding the use of renewable energy [3].

Biomass and municipal waste are interesting sources of renewable energy [4,5,6,7,8]. However, there are still unsolved problems related to the thermochemical conversion processes for these feedstocks in energy generation. Biomasses and waste are known for their different ash systems compared to coals; biomasses and waste are characterized by higher amounts of volatile species. This is accompanied by a higher risk of deposit formation, which causes a lower plant performance due to corrosion and reduced heat transfer [5,9]. From an economic point of view, amongst other things, a boiler can break down due to metal corrosion by aggressive ash components, such as chlorine, sulfur, or alkali metals [10,11,12,13,14]. The high temperature of the conversion process additionally accelerates the corrosion and the degradation of boiler parts, including heat exchanger tubes or combustion chamber walls. The use of common boiler steel grades is not favorable with these kinds of fuels; under these aggressive conditions, other materials must be used.

In order to achieve an appropriate price-to-lifetime ratio and significantly extend the life of the boiler parts, an approach based on creating cladded layers of a more corrosion-resistant material on common boiler steels is frequently applied [15,16,17,18,19,20]. The desirable materials for working in conditions that increase the risk of high-temperature corrosion include nickel-based alloys and super-alloys [16,17,18,20,21,22,23,24,25,26]. Among other things, these materials are characterized by properties such as good weldability, high creep resistance, and resistance to high-temperature corrosion. The higher price related to their composition from elements such as chromium or nickel can be considered the main disadvantage of these alloys, but in some cases, this issue can be solved by preparing a cladded layer. One interesting alternative to traditional welding processes is the method of cold metal transfer (CMT) [19,20,23,26,27,28]. In comparison to traditional MIG/MAG (metal inert gas/metal active gas) welding, the amount of heat applied to the base material (common boiler steel) is significantly lower when using CMT. That advantage plays a crucial role not only in investigating changes in the microstructure, but also in terms of the diffusion of base elements, especially iron, into the cladded layer. If the amount of iron coming from the substrate is lower in the cladded layer, the possibility of iron oxidation is lower; thus, the oxidation process is assumed to be further inhibited.

The aim of the present study is to investigate the corrosion resistance of Inconel 625 in form of CMT cladding layers on a common boiler steel. This material is prepared by a boiler company, and in this form, it may work in power plant installations. Additionally, the high temperature corrosion test environment involves the ashes obtained from renewable energy sources—oat straw ash, wood biomass ash, as well as sewage sludge ash; thus, it may more precisely imitate the conditions which occur during thermal conversion of biomass and municipal wastes. The corrosion product layer is formed by the interaction between ash and the CMT layer in conditions of high-temperature oxidation in air (combustion atmosphere). The corrosion tests are carried out at 650 °C for 1000 h in the presence of ashes from biomasses and municipal waste. The analysis of the composition and mineral phases of the corrosion products is performed by means of X-ray fluorescence (XRF), scanning electron microscopy with energy-dispersive X-ray spectroscopy (SEM–EDX), and X-ray diffraction (XRD) to identify the mechanisms of the corrosion and to evaluate the resistance of the CMT-cladded materials to ashes of different compositions.

## 2. Materials and Methods

### 2.1. Materials

This study investigated a common boiler steel grade 16Mo3 tube improved by an Inconel 625-cladded layer. The coating was prepared at the boiler manufacturing plant using the cold metal transfer (CMT) method. The Inconel CMT was cladded on the surface of 16Mo3 boiler steel (outer diameter = 60 mm, wall thickness = 6 mm). In turn, the thickness of Inconel CMT cladding was 2–3 mm. The chemical composition of the Inconel 625 is listed in Table 1. The parameters of the applied cladding process, the microstructure, and the properties of the Inconel 625 coating have been described elsewhere [13,20,26]. The pipes were cladded circumferentially in the horizontal position. Cooling with running water was used during the process. Single-layer surfacing was used. Argon was carried out as a protective atmosphere. The other process parameters were cladding current—200 A, cladding voltage—20 V, gas flow rate—17 L/pm, and peripherical speed rate—4.5 rotation/pm. Before the corrosion tests, the base material layer was removed by rolling. Pieces of 10 mm × 20 mm were cut from the tubes and the thickness of such samples was about 2.5 mm. Prior to the exposure tests, the surface was cleaned with deionized water, acetone, and ethyl alcohol. In order to investigate the form of the material which may work in real conditions (according to the recommendations of ASTM–G1-03 standard [29]), the grinding and polishing procedure was not carried out in this particular case.

Ashes of two biomasses and one waste material were produced at 550 °C in line with DIN EN ISO 18122 [30] for the subsequent exposure tests. These cover ashes from oat straw (OA) and mixed wood (WA), as well as a municipal sewage sludge (SA).

### 2.2. Methods

#### 2.2.1. X-ray Fluorescence (XRF) and Chemical Analysis

Prior to the corrosion tests, the chemical composition of the ashes was obtained by X-ray fluorescence (XRF), which was performed in a Bruker S8 Tiger (Bruker Corporation, Billerica, MA, USA) using Rh radiation in line with the standard DIN 51729-10. This composition was given in the form of oxides due to ashing in air. To determine the residual carbon content in the ash, elemental analysis was carried out using the vario EL cube (type, Heraues, Franfurkt, Germany) from Elementar Analysen systeme in line with the standard DIN 51733 [31].

#### 2.2.2. Long-Term Exposure Tests

For the long-term exposure tests, the cleaned metal samples were covered by the respective ash and were placed in an oven at 650 °C for 1000 h for corrosion tests regarding the ASTM–G1-03 standard. Ashes of two different biomasses and sewage sludge were applied in this study.

#### 2.2.3. Analyses of the Samples after the Exposure Tests

The samples of Inconel 625 were investigated on the surface and cross-section. After the corrosion test, the remaining ashes were removed from the surface, and polished sections were fabricated from the treated metals.

The phase composition of the corrosion product layer at the surface of the sample was analyzed in the near-surface area by means of X-ray diffraction (XRD) in a Bragg–Brentano configuration using a PANanalytical EMPYREAN DY 1061 (Malvern, Worcestershire, UK). For this purpose, Cu K_α_ radiation (λ = 0.1554 nm) was employed.

After the corrosion tests, the ash particles, and the surfaces and cross-sections of metal samples, were investigated by means of scanning electron microscopy (SEM) equipped with energy-dispersive X-rays (EDX), performed with an FEI Inspect S50 (FEI Company, Hillsboro, OR, USA) and an FEI Nova NanoSEM 450 (FEI Company, Hillsboro, OR, USA). The images were obtained at acceleration voltages of 15–25 kV (spot between 4–5). Element maps of the cross-sections’ near-surface were recorded using this technique at acceleration voltages of 25 kV (spot 4.5).

## 3. Results and Discussion

### 3.1. Analyses of the Ashes Prior to the Exposure Tests

Table 2 lists the results for the chemical composition of the ashes, which were subsequently used for the long-term exposure tests, obtained by X-ray fluorescence (XRF), as well as calculated B/A (base to acid) ratio; the equations have been described elsewhere [11]. All the ashes demonstrated characteristic chemical compositions for the particular type of fuel [4,5,8]. The mixed wood ash (WA) was rich in CaO and K_2_O, followed by SiO_2_ and MgO, which is a typically basic ash (B/A = 3.3) for wood and woody biomass. The high content of CO_2_ was caused by the CaO, which led to the formation of CaCO_3_ at the ashing temperature. Meanwhile, the oat straw ash (OA) was rich in K_2_O and SiO_2_, which is common for straw with an intermediate ash system (B/A = 1.1). In both ashes, an increased phosphor content was identified. The main components of sewage sludge ash (SA) are P_2_O_5_, SiO_2_, CaO, Fe_2_O_3_, and Al_2_O_3_, with a characteristically acid ash system (B/A = 0.5). Based on the studies by Vassilev et al. [4,5,8], it is concluded that these samples are representative for their particular fuel group. Additionally, a comparison between those groups of biomasses demonstrates different main ash compositions, i.e., that wood and woody biomass are rich in CaO and MgO, while straws are enriched in K_2_O, SiO_2_, and Cl. Wood and woody biomasses are frequently low in Cl, SiO_2_, and SO_3_, while straws are depleted in Na_2_O_4_. The composition of sewage sludge may not only vary significantly within different areas, but may also change over time, depending on current consumption trends. The main ash system for this kind of waste is widely determined by SiO_2_–P_2_O_5_–Fe_2_O_3_–Al_2_O_3_–CaO [32]. Moreover, some other heavy metals, such as Zn, As, Cd, Cr, Mo, Ni, Hg, or Pb, can be found in the sewage sludge and/or its ash [33,34,35].

### 3.2. SEM Analysis of Inconel Surface after Exposure Tests

**Figure 1:**
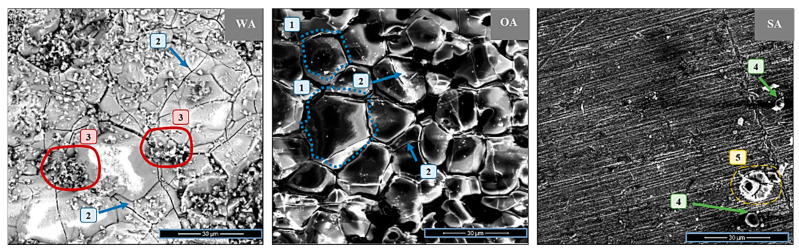
SEM images of an Inconel 625 surface after corrosion tests with mixed wood ash (WA), oat straw ash (OA), and sewage sludge ash (SA).

The surfaces of the metal samples after the corrosion tests are displayed in Figure 1. After exposure to wood and straw ashes at high temperature, the surfaces of both samples showed corrosion products and fractures of the CMT surface. Inconel 625 claddings tested with WA exhibited scaly spalling structure (marked by the number 2) and areas of clustered particles (number 3), while a distinct grain boundaries (marked by dotted lines and the number (1)) was present for OA. The corrosion product layer was not continuous and exhibited constrictions/fractures (marked by the number 2 for OA). At the edges of the WA clusters, particles of irregular shape could also be identified (3). This may have resulted from residual ash or reaction/corrosion products, which the mechanical treatment could not remove after heating at a high temperature. Significantly different results were obtained for samples after tests for corrosion by the biomass ashes (WA, OA) compared to those exposed to sewage sludge ash (SA). No distinct structure of grain boundaries and spalling were found for exposure to SA; only melted ash residues (4) and some irregularly shaped particles (5) could be distinguished.

### 3.3. Mineral Phase Analysis after Exposure Tests

**Figure 2:**
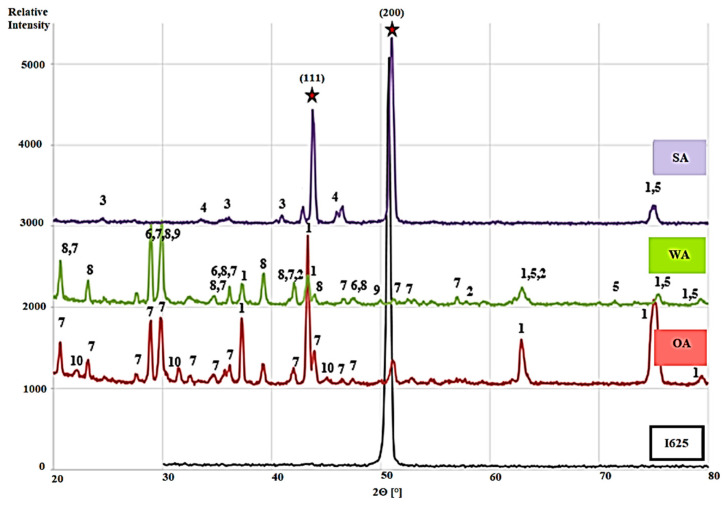
The XRD phase analysis of pure Inconel 625 (I625) and after exposure to the three ashes (OA, WA, and SA) with the identified mineral phases: 1—NiO, 2—Fe_3_O_4_, 3—Fe_2_O_3_, 4—Cr_2_O_3_, 5—NbO, 6—CaCO_3_, 7—K_2_CrO_4_, 8—K_2_MnO_4_, 9—K_2_MoO_4_, 10—KNbO_3_, ★—the signal coming from the substrate, austenitic matrix plane 2θ = 43.1° (111), 2θ = 50.3° (200).

An X-ray diffraction analysis of the surface area was performed to confirm the occurrence of corrosion products and layers, and to distinguish the mineral phases formed during the corrosion tests with three different ashes from that of the pure material (visualized in Figure 2). The measurements were carried out in a Bragg–Brentano configuration, which guaranteed that the sample was probed near to the surface. Thus, the appearance of substrate-related peaks indicated that the corrosion layer was thin. This was observed particularly in the case of sewage sludge ash (SA). The most intense peak of the substrate, at an angle of 2θ = 51–52°, was observed for all samples exposed to ash. The oxides of the substrate material were detected, such as nickel oxide, niobium oxide, and iron oxide, as well as additional compounds resulting from the reaction between the substrate components and ash components. These were mainly induced by the presence of potassium in the ashes of WA and OA; the mineral phases potassium chromate, potassium manganate, potassium molybdate, and potassium niobate were ascertained. Calcium carbonate, a residue from the ash without any reaction with the metal, was observed after the exposure tests using the WA ash.

Similar mineral phases were detected in other studies with biomass ashes by Mlonka-Mędrala et al. [12] when carrying out corrosion experiments on different grades of steel with ashes. It was confirmed that the generation of potassium chromate was the main corrosion mechanism [12]. However, it was revealed that the steel oxidation rate decreased with a higher chromium content [12]. Solecka et al. [13] investigated Inconel 625 exposed to ash rich in CaO and SiO_2_ over a long period. It was concluded that oxides generated during the experiment exhibited a two-layer structure, i.e., the outermost layer consisted of NiO, whereas coherent grains of NiCr_2_O_4_ were found at the bottom of the NiO region. In that region, a fine-grained layer composed of a mixture of NiCr_2_O_4_ and Cr_2_O_3_ was present below the coarse one. In the work of de Sousa Malafaia et al. [25], the authors examined Inconel 625 at a high temperature. After oxidation, a chromium oxide layer was observed and spinel was additionally detected due to the manganese oxidation at 1000 °C. Comparing the results obtained by other authors [12,25] and in this study, the compounds mentioned above, especially potassium chromate or nickel and chromium oxides, were formed during exposure tests and were assumed to be the main corrosion mechanisms.

### 3.4. SEM Analysis of Inconel Sample Cross-Section after Exposure Tests

**Figure 3:**
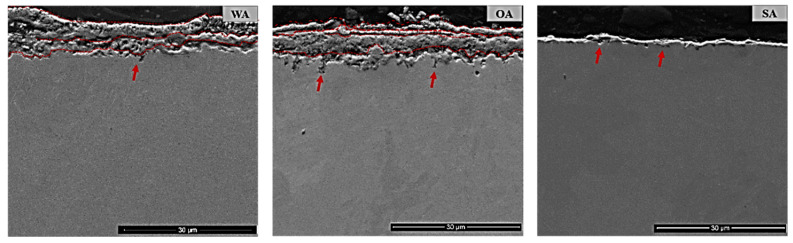
SEM images of cross-sections of Inconel 625 after exposure to the mixed wood biomass ash (WA), oat straw ash (OA), and sewage sludge ash (SA).

The cross-sections of the Inconel sample exposed to the three ashes are displayed in Figure 3. A corrosion product layer formed in the presence of the two biomass ashes (OA, WA) was significantly thicker than that observed after exposure to sewage sludge ash. For both biomass ashes, the layer thickness reached about 15 µm. In general, the layer was inhomogeneous and cracked, and the thickness varied over the sample between approx. 5 µm and 15 µm. The corrosion products did not form one compact layer: separate layers could be distinguished (marked by dashed lines in Figure 3). In contrast to this, the sample exposed to sewage sludge ash exhibited a layer thickness of 1–5 µm. This layer was also inhomogeneous and cracked in some places. In addition, there were certain areas on the surface where the corrosion layer was not observed, or it may have fallen off during the sample preparation, indicating the layer’s fragility. Pitting corrosion (marked by red arrows) was observed for all samples, but was higher for exposure to OA and WA in comparison to SA.

**Figure 4:**
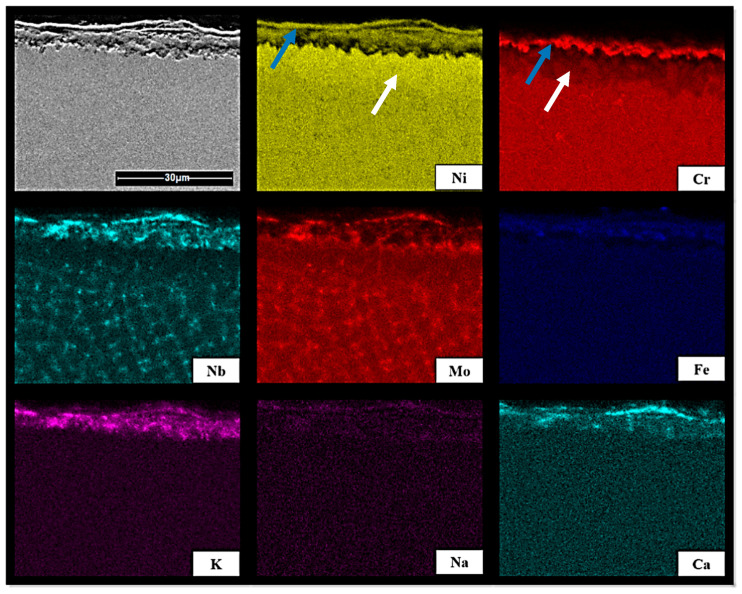
The SEM–EDX map of element distribution of Inconel 625 after exposure to mixed wood biomass ash (WA).

**Figure 5:**
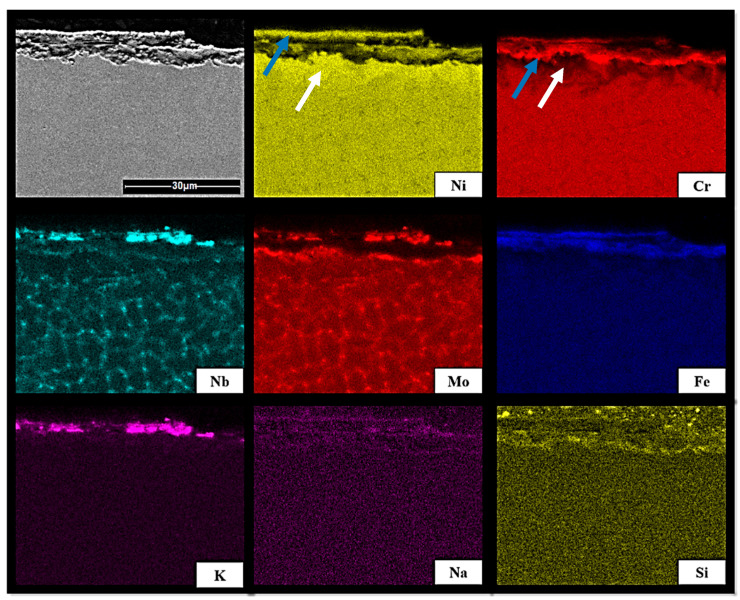
The SEM–EDX map of element distribution of Inconel 625 after exposure to oat straw ash (OA).

The determination of the mechanism leading to the formation of the corrosion product layer played a key role in the process of understanding the corrosion mechanism. For this reason, the cross-sections of the samples were investigated by means of SEM–EDX mapping. The obtained element distributions within thick corrosion layers are visualized for the sample of Inconel 625 after exposure to the mixed wood biomass ash (WA) in Figure 4, and to oat straw ash (OA) in Figure 5. In the case of exposure to WA, it was noticed that nickel and niobium were enriched at the outer surface of the corrosion layer compared to the depleted bulk material, and chromium was concentrated between the bulk and corrosion layer (enrichment indicated by blue arrow and depletion by white). Similar observations were made by Solecka et al. [13]. Here, mixtures of oxides were observed with areas rich in elements such as Cr or Ni, as well as others with lower concentrations. The related diffusion of the elements, such as chromium or nickel, was directed towards the surface, where the oxidation process took place [13]. As a result, the region beneath the oxidation zone was depleted of those elements. It was assumed that the corrosion of Inconel 625 was caused by three different mechanisms [13]: (1) in the outer layer, large crystals of nickel oxide form; (2) in the lower part of the coarse-crystalline area, the corrosion was provoked by spinel formation (NiCr_2_O_4_); and (3) underneath, there was a fine-crystalline layer of NiCr_2_O_4_ spinel mixed with Cr_2_O_3_, which subsequently continued in an inner layer built of a fine-crystalline hetero-phase mixture of Cr_2_O_3_, NiCr_2_O_4_, and NbCrO_4_. This layer can be considered as the true protective barrier (cf. distribution of K in Figure 4 and Figure 5). However, the corrosion tests carried out on Inconel with a CMT-cladded layer used an ash mainly composed of CaO and SiO_2_, while the content of potassium was only 1.5 wt% in this study [13]. Thus, in the presented study, the formation of K_2_CrO_4_ in case of exposure to K-rich agricultural and woody biomass ashes was preferred.

Interesting observations were made for the heterogeneous pattern for the distribution of elements such as Nb and Mo in Figure 4 and Figure 5. The findings for the investigation in the ash atmosphere were in a good agreement with the results obtained by Rozmus-Górnikowska et al. [20], Solecka et al. [26], and de Sousa Malafaia et al. [25] for Inconel samples without the oxidation or with oxidation in the air atmosphere. All of these authors concluded that there was a significant enrichment of interdendritic areas in Nb and Mo (for Mo the effect was lower compared to Nb), the phases rich in niobium and molybdenum formed at grain boundaries. The distribution of Cr, Ni, and Fe in the deposit was quite even. The equilibrium partition coefficient k for Mo and Nb was less than 1, thus, during the crystallization process, these elements segregated into liquids. In conclusion, after crystallization, the interdendritic areas were significantly enriched in Mo and Nb [20].

## 4. Conclusions

An investigation was carried out into the corrosion caused by different biomass and waste ashes on an Inconel 625-cladded layer prepared using the cold metal transfer (CMT) method. Based on the analysis performed, the following general conclusions can be drawn:

All ashes have demonstrated corrosion effects on the Inconel CMT-cladded samples. However, the thickness and the composition of the corrosion layer was heterogeneous over the investigated surface section. From the cross-sections, the thickness of the layers was determined to be in the range of approx. 1–15 µm, depending on the ash used during the experiment. The biomass ashes formed thick corrosion layers (approx. 5–15 µm), while the sewage sludge ash caused a thin layer (approx. 1–5 µm). 

The main mineral phases determined at the near-surface area could be roughly divided into those caused on the one hand by oxidation (e.g., NiO, Fe_3_O_4_, Fe_2_O_3_, Cr_2_O_3_, and NbO), and on the other, primarily by the interaction between the potassium from the ashes with the oxidized metal (e.g., K_2_CrO_4_, K_2_MnO_4_, K_2_MoO_4_, and KNbO_3_). The outermost layer was predominantly composed of nickel compounds. Thus, the thickness of the corrosion layer could be directly related to the elevated concentrations of potassium in the ashes of mixed wood and oat straw compared to sewage sludge.

The corrosion involved different mechanisms, from the diffusion of Ni and Cr, to the oxidation zone at the outer surfaces. The Cr was enriched in the corrosion layer and Ni was highly concentrated at the outer layer of the bulk material, while Cr and Ni were depleted in the bulk material. Finally, the corrosion of the bulk material was stopped by the formation of NiCr_2_O_4_ spinel mixed with Cr_2_O_3_ and a fine-crystalline hetero-phase mixture of Cr_2_O_3_, NiCr_2_O_4_, and NbCrO_4_. Thus, the main factors behind the corrosion process were firstly, the typically high process temperatures over a long period of continuous plant operation; secondly, the presence of corrosive agents in the ashes, such as potassium, generated from biomass and waste; and thirdly, the progress of the oxidation-induced corrosion up to the depth at which a spinel layer forms.

## Figures and Tables

**Table 1:** Chemical composition of Inconel 625 (prior to the corrosion tests).

Element	C	Si	Mn	Cr	Ni	Mo	Nb	Ti	Fe
Mass wt %	0.1	0.4	0.4	21.0–23.0	By balance	8.0–10.0	3.2–3.8	0.4	<1.0

**Table 2:** Chemical composition of the investigated ashes determined after ashing in line with DIN EN ISO 18122 and DIN 51733.

Composition/wt%	Mixed Wood Ash (WA)	Oat Straw Ash (OA)	Sewage Sludge Ash (SA)
CO_2_	19.80	7.00	0.00
Na_2_O	0.00	0.90	0.53
MgO	8.29	1.98	5.86
Al_2_O_3_	1.89	0.86	7.55
SiO_2_	8.57	33.27	21.00
P_2_O_5_	7.24	9.67	29.09
SO_3_	1.29	2.04	1.85
Cl	0.05	0.57	0.00
K_2_O	15.18	36.80	2.26
CaO	34.84	6.47	20.55
TiO_2_	0.05	0.05	0.97
Mn	1.91	0.10	0.08
Fe_2_O_3_	0.90	0.28	10.27
Sum	100.0	100.0	100.0
B/A (mass basis)	3.28	1.05	0.50
